# Macroevolutionary Analyses Suggest That Environmental Factors, Not Venom Apparatus, Play Key Role in Terebridae Marine Snail Diversification

**DOI:** 10.1093/sysbio/syz059

**Published:** 2019-09-05

**Authors:** Maria Vittoria Modica, Juliette Gorson, Alexander E Fedosov, Gavin Malcolm, Yves Terryn, Nicolas Puillandre, Mandë Holford

**Affiliations:** 1 Department of Biology and Evolution of Marine Organisms, Stazione Zoologica Anton Dohrn, Villa Comunale, 80121 Naples, Italy; 2 UMR5247, Université de Montpellier CC 1703, Place Eugène Bataillon 34095 Montpellier, France; 3 Department of Chemistry, Hunter College Belfer Research Center, 413 E. 69th Street, BRB 424, New York, NY 10021, USA; 4 Department of Biochemistry, Weill Cornell Medical College, Cornell University, New York, NY 10021, USA; 7 Institute of Ecology and Evolution of Russian Academy of Sciences, Leninskiy Prospect, 33, Moscow 119071, Russia; 8 Bird Hill, Barnes Lane, Milford on Sea, Hampshire, UK; 9 Institut Systématique Evolution Biodiversité (ISYEB), Muséum national d’Histoire naturelle, CNRS, Sorbonne Université, EPHE, Université des Antillles, 57 rue Cuvier, CP 26, 75005 Paris, France

**Keywords:** Terebridae, macroevolution, phylogenetic comparative methods, venom, Conidae, diversification

## Abstract

How species diversification occurs remains an unanswered question in predatory marine invertebrates, such as sea snails of the family Terebridae. However, the anatomical disparity found throughput the Terebridae provides a unique perspective for investigating diversification patterns in venomous predators. In this study, a new dated molecular phylogeny of the Terebridae is used as a framework for investigating diversification of the family through time, and for testing the putative role of intrinsic and extrinsic traits, such as shell size, larval ecology, bathymetric distribution, and anatomical features of the venom apparatus, as drivers of terebrid species diversification. Macroevolutionary analysis revealed that when diversification rates do not vary across Terebridae clades, the whole family has been increasing its global diversification rate since 25 Ma. We recovered evidence for a concurrent increase in diversification of depth ranges, while shell size appeared to have undergone a fast divergence early in terebrid evolutionary history. Our data also confirm that planktotrophy is the ancestral larval ecology in terebrids, and evolutionary modeling highlighted that shell size is linked to larval ecology of the Terebridae, with species with long-living pelagic larvae tending to be larger and have a broader size range than lecithotrophic species. Although we recovered patterns of size and depth trait diversification through time and across clades, the presence or absence of a venom gland (VG) did not appear to have impacted Terebridae diversification. Terebrids have lost their venom apparatus several times and we confirm that the loss of a VG happened in phylogenetically clustered terminal taxa and that reversal is extremely unlikely. Our findings suggest that environmental factors, and not venom, have had more influence on terebrid evolution.

Explaining the amazing biodiversity of species that inhabit our planet remains a significant challenge. With the exception of a few well-known taxa, such as vertebrates or angiosperms, current hypotheses about diversity patterns remain largely untested across the majority of Earth’s biodiversity (Jetz et al. 2012; Pyron and Burbrink 2012; Rainford et al. 2014; Legendre and Condamine 2018). This is especially true for marine invertebrates, in which their basic biology, diversification patterns, and evolutionary dynamics remain largely unknown. Several hypotheses proposed to explain diversity patterns focus on key innovations that affect the adaptation of organisms to their environment. The innovations can be derived from intrinsic factors like morphology, physiology, behavior, ecology, or from extrinsic environmental factors, such as depth and temperature (Benton and Harper 2009; Yoder et al. 2010; Ng and Smith 2014; Wiens 2017). The acquisition of key innovations is proposed to lead to faster diversification rates either by increasing speciation rates or by decreasing extinction rates, which may account for differences in species richness between clades (Rabosky et al. 2013; Rainford et al. 2014; Sánchez-García and Matheny 2017). In addition, environmental modifications may create new ecological opportunities for specific clades, through the availability of new habitats or the extinction of predators or competitors (Harmon et al. 2008; Parent and Crespi 2009; Des Roches et al. 2011).

Many marine organisms rely on the production of venomous secretions to deter predators or subdue preys. The onset of a venom system, made up of specialized glands and delivery structures such as beaks, fangs, harpoons, spines, or pincers, is considered an opportunistic innovation that favors speciation of predators by enabling the exploitation of new ecological niches characterized by different potential prey species (Vidal and Hedges 2005; Fry et al. 2006; Castelin et al. 2012). Venom plays a crucial role in prey capture and survival, which makes it a potential key innovation, as also suggested by its convergent evolution in multiple lineages (Barlow et al. 2009; Casewell et al. 2013). The components of venom are often encoded by rapidly evolving gene families (Kordis and Gubensek 2000; Fry et al. 2009; Casewell et al. 2013), suggesting a strong diversifying selective pressure on venom composition. However, the hypothesis that venom production may affect diversification has only been examined in a few cases, mostly in vertebrates or terrestrial invertebrates and is generally targeted at the species level using indirect evidence (Daltry et al. 1996; Fry et al. 2008; Duda et al. 2009). For example, in snakes, which exhibit exceptional species richness, it is proposed that the majority of the diversity stems from an early radiation within the superfamily Colubroidea, possibly due to the evolution of venom delivery systems that allowed the colonization of new areas (Pyron and Burbrink 2012).

Marine snails belonging to the superfamily Conoidea are among the most prominent marine venomous lineages. To date extensive toxinological and phylogenetic investigations have focused almost exclusively on *Conus* species, neglecting other related lineages, including the Terebridae or auger snails (Holford et al. 2009a; Puillandre et al. 2011; Castelin et al. 2012). Terebrids demonstrate a high level of morphological disparity in feeding-related traits, in shell size range, and ecological diversity, providing a basis for investigating the role of such traits as diversification drivers. The more than 400 described terebrid species display anatomical disparity in the foregut comparable with the entire Conoidea superfamily (Miller 1971; Mills 1979; Castelin et al. 2012). The terebrid foregut has been shaped by multiple losses of key anatomical structures such as the venom gland (VG) and proboscis, as well as by the convergent evolution of the main venom delivery structure, the hypodermic radula, in three lineages (Castelin et al. 2012). Given this remarkable variation, we examined if morphological traits pertaining to the use of venom may have affected terebrid evolution.

Recognizing that not all terebrids have a venom apparatus, we also examined the role of additional biotic and abiotic traits pertaining to shell size, larval ecology, and depth in driving diversification of the Terebridae. Similar to foregut anatomy, shell size displays a high level of diversification in Terebridae, which in adult specimens can range from 15 to 230 mm (Taylor 1990; Terryn 2007; Terryn and Holford 2008). Body size influences multiple aspects of organismal morphology, physiology, life history, and ecology, and may dramatically affect behavior and extinction rates. The relationship between body size and diversification rates is mostly unresolved and has been confirmed only in a few cases (Knouft and Page 2003; Fontanillas et al. 2007; Rabosky et al. 2013). However, most studies failed to identify a clear effect of size on lineage diversification (Gittleman and Purvis 1998; Owens et al. 1999; Rainford et al. 2014; Feldman et al. 2016; Lee et al. 2016). In terebrids, diversification of shell size might both affect speciation rates allowing access to multiple trophic niches and influence the extinction risk through a balance between the higher metabolic expenditure and the differential susceptibility to predation.

As in other marine gastropods, terebrids can produce pelagic larvae that either actively feed on phytoplankton (planktotrophy) or rely exclusively on yolk reserves (lecithotrophy; Thorson 1950). Although planktotrophic larvae can spend a considerable time in the water column (typically weeks or months), lecithotrophic larvae have a shorter pelagic phase due to the limited yolk reserve and consequently tend to have reduced dispersal kernels (Shanks 2009). The duration of pelagic larval phase has been demonstrated to influence genetic connectivity in gastropods (Collin 2001; Wright 2002; Modica et al. 2017), through dispersal ability, suggesting that the acquisition of lecithotrophy may lead to increased speciation rates by reducing gene flow between populations (Harvey et al. 2017).

A relationship has been proposed between diversification and abiotic factors such as habitat complexity, sea temperature, sea level, ocean productivity, and oxygen content, for different lineages of marine organisms (Figueirido et al. 2011; Stein et al. 2014; Davis et al. 2016; Costello and Chaudhary 2017; Stigall 2017; Lewitus et al. 2018; Rabosky et al. 2018). Indeed, depth has been identified as a diversification driver in several lineages of marine fish (Ingram 2011; Sorenson et al. 2014; Gaither et al. 2016). Given terebrids have a broad span of bathymetric distribution globally in subtropical and tropical oceans, where they have been found on the shore line as well as at depths $>$700 m (Taylor 1990; Terryn 2007; Terryn and Holford 2008), depth is another important factor to investigate for influence on terebrid diversification.

In this study, we reconstruct the first dated terebrid phylogeny with a three-fold increase in number of specimens analyzed from prior efforts and use this tree to carry out a phylogenetic comparative analysis of morphological and life-history traits, along with bathymetric distribution, and their association to diversification regimes in terebrid marine snails (Fig. 1). We separately evaluate support for the hypothesis that the venom apparatus, shell size, larval development, and depth have facilitated diversification in marine snails.

**Figure 1.**
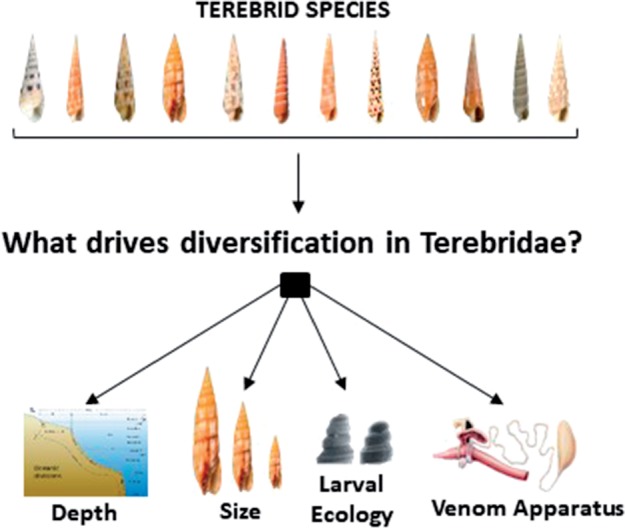
Examination of environmental, ecological, and morphological traits to determine factors driving evolution and diversification in the Terebridae. One thousand seven hundred and sixty-one (1761) specimens of Terebrids were collected globally and sequenced using a multigene strategy to reconstruct a phylogenetic hypothesis that was dated using input from the fossil record, and subsequently used to infer diversification patterns for the family. Disparities in size, larval ecology, depth, and presence or absence of the venon gland were evaluated to determine their impact on terebrid diversification rates.

## Materials and Methods

### Sample Collection

All of the materials used in this study were collected during several expeditions conducted by the Museum National d’Histoire Naturelle of Paris (MNHN–www.expeditions.mnhn.fr) and the Holford Laboratory. The data set includes 1275 specimens collected from 25 localities with a focus on the Indo-Pacific province (Supplementary Table S1 available on Dryad at http://dx.doi.org/10.5061/dryad.j008j5h). Samples were collected from 0 m to approximately 800 m in depth and specifically fixed for molecular analysis in the field. Live specimens were anesthetized using magnesium chloride (MgCl$_{2})$ isotonic with seawater, and a piece of tissue was cut from the foot and fixed in 95% ethanol. Specimens collected after 2012 were processed with a microwave oven to facilitate removal of soft tissue from the shell (Galindo et al. 2014). The majority of shells was kept intact for identification and deposited as vouchers in MNHN and the Holford laboratory. The taxonomy of the family Terebridae was reworked based on the new phylogeny provided in this study. The nomenclature for new taxa and revised classification of Terebridae based on the recently portrayed relationships is followed (Fedosov et al. 2019).

### DNA Sequencing and Molecular Phylogenetic Analyses

Total genomic DNA was extracted from foot tissue using NucleoSpin® 96 Tissues (Macherey-Nagel) or the Epmotion 5075 robot (Eppendorf), following the manufacturer’s protocol. Fragments of three mitochondrial genes [Cytochrome Oxidase I (COI), 16S rRNA, and 12S rRNA] and one nuclear gene (28S rRNA) were amplified. PCR reactions were performed as described in Holford et al. (2009a). Successfully amplified products were sent to Genewiz (South Plainfield, NJ, USA) or to the Eurofins sequencing facility (France) for bidirectional Sanger sequencing.

Sequences were aligned for each gene independently using MUSCLE version 3.2 (Edgar 2004). The accuracy of these alignments was manually inspected using BioEdit version 7.0.0.0 (Hall 1999). Best-fit substitution models were identified for each gene separately using jModelTest2 version 2.1.6 (Posada 2008). Best-scoring maximum likelihood (ML) trees were estimated using RAxML (Stamatakis 2006, 2014). Each gene, and each codon position within the COI gene, was considered as independent, each following its best-fit substitution model. Robustness of the nodes was assessed using the thorough bootstrapping algorithm (Felsenstein 1985) with 1000 replicates. Phylogenies were jointly estimated using the Bayesian Markov Chain Monte Carlo method implemented in BEAST version 1.8.4 (Drummond and Rambaut 2007). The program BEAUti version 1.8.4 (Drummond and Rambaut 2007) was used to generate the file used in BEAST. A birth–death process speciation prior was implemented and the substitution models identified in jModelTest2 version 2.1.6 were applied to each gene independently. An uncorrelated lognormal clock was applied to estimate the relaxed molecular clock. The analysis ran for 75 million generations and sampled every 1000 generations. The oldest known Terebridae, *Mirula plicata* (Lamarck, 1803) from the lower Eocene (56.0–47.7 Ma) was used to constrain the stem node of Terebridae with a normal distribution mean of 50.7 Ma and a standard deviation (SD) of 1.48 (Abdelkrim et al. 2018). A burn-in of 10% was removed after convergence analysis was evaluated using Tracer version 1.7 (Drummond and Rambaut 2007) to check that all effective sample size (ESS) values were $>200$. Analyses were performed on the Cipres Science Gateway (http://www.hylo.org/portal2), using the RAxML-HPC2 on XSEDE tool for ML and the BEAST on XSEDE tool for BA.

### Shell Size Measurements

Shell sizes were determined for 325 intact adult specimens representing 137 species of our data set. Reliability and species-level representativeness of these measurements were checked against size ranges published by Bratcher and Cernohorsky (1987) or in the original descriptions of the shells. For trait-dependent diversification analyses, shell size was converted into a binary categorical trait with the states “small” and “large,” following a shell size partitioning that was obtained as follows: From the species present in our DNA data set, we calculated the lowest 25% quartile for species size and adopted 25 mm, which accommodated 30% of the species, as the boundary for the categorical size trait with the states of “small” or “large” for each species. Each measurement was confirmed against published information regarding shell size to ensure that the allocation to the small or large species category was reasonably valid.

### Larval Ecology

In Terebridae, as in many other families of marine gastropods, larval ecology can be easily inferred from the appearance of protoconch, the larval shell that is often maintained at the tip of adult shell (Jablonski and Lutz 1983; Lima and Lutz 1990; Eldredge et al. 2005). Depending on the protoconch appearance, species are defined as planktotrophic, that is possessing a pelagic free swimming stage during which the veliger larva can actively collect phytoplankton, when the protoconch is multispiral, or lecithotrophic, relying on yolk reserves for survival until metamorphosis (Thorson 1950), when the protoconch is paucispiral. The protoconchs of 638 intact terebrid shells were examined under a microscope and categorized as multi- or paucispiral, and the number of whorls present was counted to the nearest quarter whorl (Bouchet and Kantor 2004).

### Foregut Anatomy

The anatomy of the terebrids was studied by manual dissections—when possible, on the same specimens sequenced for phylogeny. As most informative morphological characters in Conoidea are related to feeding, we specifically focused on the anterior alimentary channel structures to infer ability of the Terebridae lineages to envenomate their preys. Manual dissections were complemented by SEM studies of radular morphology, known to be extremely diverse in the Terebridae. When present, radular sacs were isolated, and soft tissues immersed in a 3–5% solution of commercially available bleach. The radulae were then rinsed several times in distilled water, mounted on a 12-mm SEM stub, air-dried, gold-coated, and examined using a TeScan TS5130MM microscope at the Joint Usage Center “Instrumental methods in ecology” at the Institute of Ecology and Evolution of Russian Academy of Sciences (IEE RAS).

### Bathymetric Distributions

To calculate the bathymetric range for each species, all the individual specimens had a depth range recorded at the time of collection giving the maximum and minimum depth of the dredge/dive at its collection station. If a station was sampled at a constant depth, the same depth value was adopted as both the maximum and minimum depth for the specimen. For each species with multiple specimens recorded, we adopted a minimum depth for the species based on the lowest maximum depth at any collecting station for a specimen of that species. This approach allowed us to be certain that at least one specimen of the species was found at that depth or shallower. Likewise a maximum depth for the species was adopted based on the highest minimum depth of all the specimens of the species. The resulting range of depth can, therefore, be considered as a reliable but a minimal value. This algorithm was implemented in an in-house Python script to quickly analyze large data sets of species occurrences (Supplementary Fig. S1 available on Dryad). For trait-dependent diversification analyses, depth was converted into a binary categorical trait with the two states “shallow” and “deep” using a 100-m threshold. The use of this depth threshold value roughly corresponds on average to the end of the photic zone and is in agreement with previous publications on marine gastropods, and represents a zone for which it is generally observed a drop in the number of collected samples due to technical limitations (Bouchet et al. 2008, 2009).

### Species Delimitation and Species Diversity Estimations

All samples were first identified morphologically. Then, independent gene trees were used to confirm that conspecific samples were all included in a single clade, separated by genetic distances compatible with intraspecific distances (i.e. inferior to genetic distances among species).

To estimate total Terebridae diversity, we used the Chao1 estimator (Gotelli and Chao 2013):

\begin{align*}\mbox{S}_{\mbox{Chao1}} =\mbox{S}_{\mbox{obs}} + f_1^2 /\left(2 f_2\right)\end{align*}

where S$_{obs}$ is the observed species richness, and f$_{1}$ and f$_{2}$ the number of, respectively, singletons (species found only once in the study area) and doubletons (species found twice).

As the overall sampling effort has been uneven with respect to the worldwide distribution of Terebridae, we used a two-steps strategy to estimate global Terebridae biodiversity. First, we calculated the S$_{Chao1}$ for the Indo-Pacific subset of our Terebridae data set, because it corresponds both to a biodiversity hotspot for molluscan fauna and to the most densely sampled area, obtaining the estimated Indo-Pacific diversity. We then calculated the ratio of the estimated Indo-Pacific diversity to the sampled Indo-Pacific diversity, a measure of how well our sampling reflects the real diversity for that specific area. Assuming that the effectiveness of our sample is the same worldwide (which is reasonable given that both diversity and sampling effort are lower outside the Indo-Pacific), we applied the same ratio to the total number of Terebridae species described in WoRMS (WoRMS Editorial Board 2018). Finally, we added to the estimate the number of newly delimited species from this study, to derive the total estimated Terebridae biodiversity. The same approach was applied to estimate the number of Terebridae species presenting alternate character state for depth, size, and larval ecology, except that the ratio was calculated between the number of Indo-Pacific species presenting, for example state 0 and the total number of Indo-Pacific species for which we had available information (state 0 + state 1). The ratio relative to state 0 and state 1 was then applied to the total Terebridae diversity estimated as described above.

### Diversification Rates through Time and Across Clades

Macroevolutionary dynamics of diversification were modeled across the Terebridae phylogeny (after outgroup removal) using the software Bayesian Analysis of Macroevolutionary Mixtures (BAMM) v.2.5.0 (Rabosky et al. 2013; Rabosky et al. 2014) on the Maximum Clade Credibility tree obtained in BEAST. BAMM explores models of lineage diversification implementing a Metropolis Coupled Markov Chain Monte Carlo (MC3) to improve the efficiency in simulating the posterior probability distribution. Ten million generations of reversible jump Markov Chain Monte Carlo sampling were run, drawing samples from the posterior every 10,000 generations. Priors were chosen using the setBAMMpriors command in the R package BAMMtools (Rabosky et al. 2014), except for the prior probability of rate shift, which has been shown to affect BAMM results (Moore et al. 2016; Rabosky et al. 2017). For this prior, we tested values ranging from 0.1 to 50 and we chose the value leading to the highest ESS values for LogLikelihood and NumberOfShifts (Supplementary Table S2 available on Dryad). We accounted for incomplete taxon sampling using a sampling fraction of 26%, estimated using a total Terebridae diversity value obtained as described above. We processed the output data using BAMMtools to obtain summary statistics after removing a 10% burn-in, and to plot diversification rate through time. BAMM was used both to estimate diversification rates through time and among/within clades, and to define diversification rates for continuous traits (depth and size) using the same parameters.

To corroborate BAMM results we used the time-dependent diversification approach implemented in the R package RPANDA (Morlon et al. 2016). This approach enables both speciation and extinction to change through time, whereas in BAMM the extinction rates are assumed to be constant, thus allowing scenarios in which diversification rates are negative (Morlon et al. 2011). For the whole Terebridae tree (with a 26% sampling fraction), we tested with RPANDA six nested diversification models: 1) a Yule model, with a constant speciation rate and null extinction, 2) a constant birth–death model, with constant speciation and extinction rates, 3) a variable speciation rate model without extinction, 4) a variable speciation rate model with constant extinction, 5) a rate-constant speciation and variable extinction rate model, and 6) a model in which both speciation and extinction rates vary (Legendre and Condamine 2018). To select the best-fitting model, ML score of each model and the resulting corrected Akaike information criterion (AICc) were compared (Supplementary Table S3 available on Dryad).

### Trait-Dependent Diversification

To model simultaneously the evolution of discrete traits and their impact on diversification, we used trait-dependent diversification models, in which species are characterized by an evolving trait and their diversification follows a birth–death process in which speciation and extinction rates may depend on the trait state. We used four characters: 1) Larval ecology, where species were defined by having either a planktotrophic (0) or nonplanktotrophic (1) ecology; 2) VG, where species were defined according to either the presence (0) or the absence (1) of this structure; 3) depth, where species were defined as shallow (0) when found above 100 m or deep water (1) below 100 m; and 4) size, where species were identified as either small (0) for shell length lower than 25 mm or large (1) for lengths exceeding 25 mm. Continuous traits were transformed into categorical two-state traits using appropriate thresholds as described above. We applied the Binary State Speciation and Extinction model (BiSSE; Maddison et al. 2007) for the four two-states data sets, accounting for state-specific incomplete taxon sampling, estimated based on our data as detailed in the Supplementary Materials available on Dryad. The BiSSE model has six distinct parameters: two speciation rates, two extinction rates, and two transition rates (i.e. anagenetic change) between the trait states. Analyses were performed using the R package diversitree (Fitzjohn 2012) on the MCC tree obtained from BEAST, using the functions make.bisse to construct the likelihood functions for each model based on the data, and the functions constrain and find.mle to apply different diversification scenarios (Supplementary Table S4 available on Dryad). We used AIC to select among different models: the scenario supported with the lowest AIC was considered the best when $\Delta $AIC$>2$ and AIC $\omega > 0.5$ against other models.

### Phylogenetic Signal and Phylogenetic Diversity

We compared the phylogenetic signal of the phenotypic traits taken into consideration (venom apparatus, shell size, larval development, and depth) using different metrics for the different type of characters. For continuous traits (size and depth), we calculated Pagel’s $\lambda $ using the function phylosig in the R package Phytools: a $\lambda = 0$ indicates a trait is random with respect to phylogeny (i.e., there is no phylogenetic signal), whereas a $\lambda = 1$ is consistent with a trait that has evolved according to the Brownian motion model (Freckleton et al. 2002). For binary discrete traits (VG and larval development), we applied the $D$ statistic proposed by Fritz and Purvis (2010), using the function phylo.d in the R package caper: $D = 1$ indicates that the trait has a phylogenetically random distribution across the tips of the phylogeny (i.e., lack of phylogenetic signal), whereas $D = 0$ if the observed trait is as clumped as if it had evolved according to a Brownian motion model. Values of $D$ can also fall outside this range: $D < 0$ suggests a highly clustered trait whereas $D > 1$ suggests phylogenetic overdispersion.

We used a phylogenetic diversity (PD) approach to measure how functional and ecological discrete traits are distributed along Terebridae phylogeny. As defined by Faith (1992), PD can be measured as “the minimum total length of all the phylogenetic branches required to span a given set of taxa on the phylogenetic tree.” In this particular context, this approach depicts how the distribution of a trait state among taxa is influenced by the underlying evolutionary processes, or in other words how each trait state contribute to the phylogenetic signal for that particular discrete trait.

PD was calculated for two subsets of taxa corresponding: 1) the planktotrophic vs. lecithotrophic developers and 2) the species with VG vs. species that had lost it. In both cases, PD was calculated using different metrics, standardized for unequal richness sampling, using the R package picante (Kembel et al. 2010, 2013). First, we calculated Faith’s PD, corresponding to the sum of the total phylogenetic branch length for one or multiple samples (Faith 1992). Then, we measured beta diversity in each subset both as the mean nearest taxon distance (MNTD) separating taxa with alternative trait states, corresponding to the average phylogenetic distance to the most similar taxon in the other cluster, and as the mean pairwise distance (MPD) separating taxa in two clusters (Gotelli and Colwell 2001; Webb et al. 2002; Helmus et al. 2007). All metrics were calculated as SES (standardized effect size) values (Warren et al. 2008). As MPD and MNTD have different sensitivity, being more sensitive, respectively, to tree-wide vs. tips-accumulating patterns of phylogenetic clustering. Positive values (mpd.obs.z $\ge 0$) and high quantiles (mpd.obs.p $> 0.95$) indicate phylogenetic evenness, or a greater phylogenetic distance among species sharing a same character state than expected. Conversely, negative values and low quantiles (mpd.obs.p $< 0.05$) indicate phylogenetic clustering, or small phylogenetic distances among species sharing a same character state than expected (Gotelli and Colwell 2001; Webb et al. 2002; Helmus et al. 2007).

### Evolutionary Modeling

To test whether shifts in larval development are associated with selective constraints on the evolution of shell size and bathymetric distribution, and if depth shifts are associated with selective constraint on shell size evolution, we fitted two Brownian Motion (BM) models and five different Ornstein-Uhlenbeck (OU) models using the R package OUwie (Beaulieu 2016) to 100 trees reconstructed with stochastic character mapping of the trait “larval development” and the trait “depth” (coded as discrete) using the make.simmap function available in the R package phytools. For the parametrization of make.simmap, we used the estimated ancestral state, and a transition matrix with equal rates estimated from our empirical data with a MCMC search, and we performed 100 replicates then summarized in a consensus tree, to account for the inherent stochasticity of the process. BM models are processes where phenotypic variation accumulates with time, as is the case with random variation, neutral genetic drift, or drift-mutation equilibrium (Felsenstein 2001; Beaulieu et al. 2012). Here, we fitted BM1 and BMS models, respectively, with a single rate and different rate parameters for each state in the tree. The OU models, add to the stochastic displacement described by BM models an optimal trait value and a tendency toward that optimum (Hansen 1997; Beaulieu et al. 2012). The simplest OU model (OU1) has a single optimum ($\theta$) applied to all branches. The remaining four OU models differ in how the rate parameters are allowed to vary in the model. In the first (OUM model) phenotypic optima means ($\theta_{x}$) are different whereas both the strengths of selection (*$\alpha $*$_{x})$ and the rate of stochastic motion around the optima (*$\sigma $*$^{2}_{x})$ acting on all selective regimes are identical. We also fitted a model that only allowed strengths of selection to vary among selective regimes (*$\alpha $*$_{1}$, $\alpha_{2}$: OUMA model), as well as one that only allowed the rates of stochastic evolution away from the optimum to vary (*$\sigma $*$^{2}_{A}$, *$\sigma $*$^{2}_{B}$: OUMV model). Eventually, we fitted a model (OUMVA) that allowed all three parameters ($\theta, \alpha, \sigma$) to vary among the different selective regimes. To choose the best-fitting model, we used a model-averaging approach, where we calculated the Akaike weights for each model, that is the relative likelihood of each model (Burnham and Anderson 2002) by means of the second-order AICc that includes a correction for reduced sample sizes (Hurvich and Tsai 1989). We ensured that the eigenvalues of the Hessian matrix calculated in our OUwie analysis were positive, because this is an indication of the reliability of parameters estimation (Beaulieu et al. 2012).

## Results

### Species Diversity Identifies Potential Cryptic Lineages

**Figure 2.**
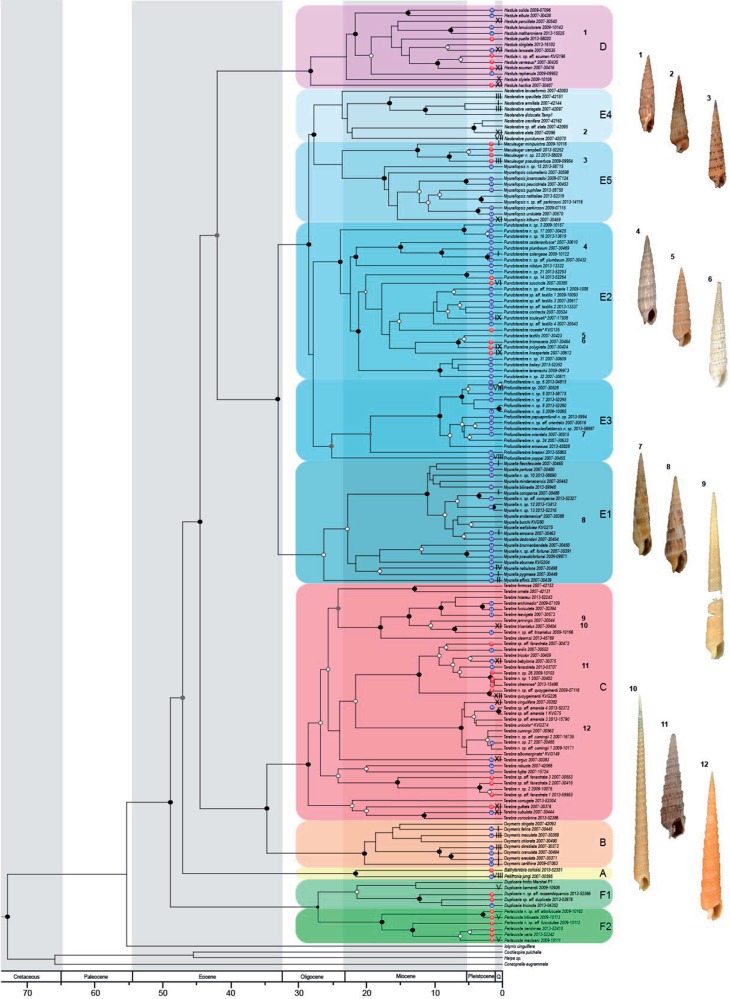
Dated phylogenetic reconstruction of the Terebridae using a multigene approach confirms terebrids are monophyletic and comprised of six major clades. A Bayesian phylogenetic terebrid tree was constructed using 12S, 16S, 28S, and COI DNA sequences. Six major Clades (A–F) were recovered, which are shown with a unique color shaded block in the tree. Each shade represents a different genera listed A–F with subheadings such as A, E1–E5, and F1, F2, within the main clades. Character traits larval ecology and anatomy types are mapped onto the tree. Blue dots with the letter “M” represent a multispiral protoconch, whereas red dots with the letter “P” represent a paucispiral protoconch. Roman numerals represent newly defined anatomy types. Shells represent 12 of the 17 cryptic species complexes identified. Posterior Probabilities (pp) are marked with dots on the nodes, where black darkened dots represent a pp of 1 and gray dots represent a pp between 0.9 and 1.0.

A data set of 1275 samples was used to reconstruct the molecular phylogeny of the Terebridae family (Fig. 2 and Supplementary Table S1 available on Dryad). Among them, 130 species were confidently identified because their shell matched a described species and corresponded to a unique lineage in the independent gene trees. Some names previously synonymized with others were elevated at the species level (marked with an * in Fig. 2; Fedosov et al. 2019). In addition, 69 new species were identified based on morphological grounds and/or correspondence to divergent lineages in the independent gene trees, with genetic distances among species equivalent or even higher to genetic distances recovered among already described species (K2P genetic distances $>$ 2.5%). For example, the name *Punctoterebra textilis* was originally applied to eight lineages recognized in the COI tree. After re-examination of the shells, we applied the name *P. textilis* to one of them, the names *Punctoterebra roseata* and *Punctoterebra soulyeti*, previously considered as synonyms of *P. textilis*, to two others, and the remaining five lineages are considered new. In all but one case taxa belonging to these species complexes fall within one major Terebridae clade consistent with one genus. The single exception is the *Profunditerebra orientalis* complex, in which two lineages cluster within the genus *Profunditerebra *(E3) and a morphologically strikingly similar form is found in *Maculauger *(E5A; Fig. 2). In most of these species complexes, a thorough re-examination of the shells revealed morphological differences, suggesting they comprise *pseudo*-*cryptic* species. Our findings suggest that a considerable fraction of the Terebridae diversity still requires formal description.

Three species complexes comprised pairs of lineages with allopatric distribution, and in three clusters comprising three or more divergent lineages (*P. textilis, Tabellaria fenestrata* and *Punctoterebra trismacaria*) where at least one of them does not overlap in distribution with others. In addition, our data suggest difference in bathymetric distribution in at least four putative species complexes: *Terebra cumingii, Myurella burchi, P. trismacaria*, and *P. orientalis*. However, such differences do not exist between sister-lineages, suggesting that the lineages within a species complex actually correspond to different species (Puillandre et al. 2012). Confirming whether these lineages correspond to different species or to populations within a single species would require further study, including more samples per lineages that are currently represented in most cases by less than five specimens each. For the subsequent analysis, we considered that our data set includes 199 species.

### Dated Terebridae Molecular Phylogeny Recovers New Sister Clade

A multigene approach was applied using COI (1161 samples), 16S (717 samples), 12S (817 samples), and 28S (263 samples) genes. Analyses of each individual gene were performed using RAxML and no supported conflicts were found between the four separately generated gene trees (Supplementary Figs. S2–S5 available on Dryad). The four genes were combined to produce a consensus tree (Fig. 2). Only samples with $\ge 2$ genes successfully sequenced were used in the combined gene data set, a total of 898 samples. Even though the species representation doubled and the number of samples tripled from the previous reported terebrid molecular phylogenies, the overall topology of the terebrid tree is largely consistent with the previous study and the family has remained monophyletic as described in the first molecular phylogeny of the group (Holford et al. 2009b).

Our new terebrid phylogenetic reconstruction divides the family into six major clades as found in previously published reports (Castelin et al. 2012). In this study, we use the same naming system for clades (A–F). However, Clade A (*Pellifronia*) is no longer a sister group to all other terebrids and two lineages were recognized, *Pellifronia jungi *and *Bathyterebra coriolisi *(Fedosov et al. 2019; Fig. 2). The genera represented by Clade B (*Oxymeris*), Clade C (*Terebra)*, and Clade D (*Hastula*) are consistent with their previous placement (Holford et al. 2009a; Castelin et al. 2012). The largest Clade E is subdivided into subclades E1–E5, with the corresponding genera E1 (*Myurella*), E2 (*Punctoterebra*), E3 (*Profunditerebra*), E4 (*Neoterebra*), and E5 (*Maculauger* and *Myurellopsis*). In addition, Clade F, consisting of 11 species in our data set, is now the sister group to all other terebrids with a posterior probability of 1. Based on morphological findings summarized in Fedosov et al. (2019), this clade has been further divided into F1 and F2, which correspond to the revised genus *Duplicaria* and the genus *Partecosta*, respectively (Fedosov et al. 2019).

We used the current fossil record of the Terebridae to produce a calibrated tree. The origin of the Terebridae is estimated at 50.6 Ma with 95% highest probability density: 44.1–51.2, matching the well-documented Terebridae fossils found in the Early Eocene period (stage Ypresian: 47.8–56 Ma). The six main lineages of terebrids all appeared before the end of the Eocene. The diversifications of each of the main lineages, including the subgroups within the Clades A, E, and F, all started concomitantly, between the mid-Oligocene (30 Ma) and the early Miocene (20 Ma).

### Terebrid Diversification Rates Increase Over Time

**Figure 3.**
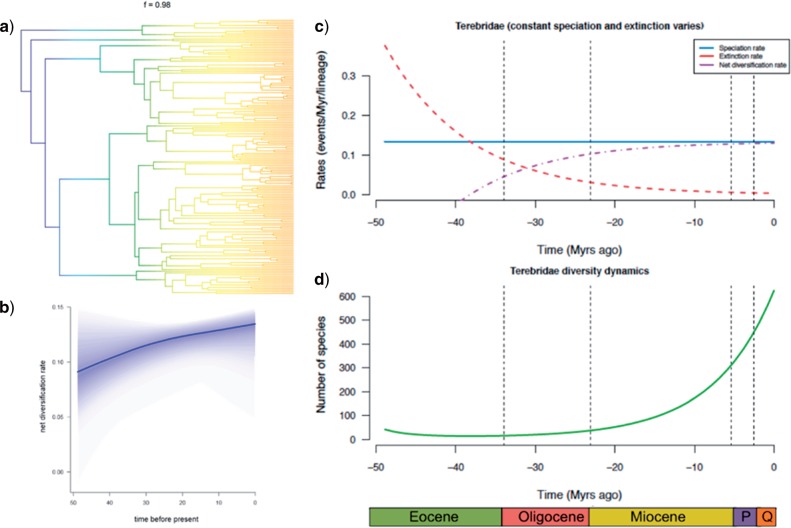
Terebridae Diversification rates vary across clades and time. a) The single BAMM credible shifts plot representing the rate shift configuration and a posteriori probability shift configuration corresponding to 0.97. b) BAMM plot depicting the net diversification rates-through-time trajectory as analyzed by BAMM. c) RPANDA plot showing the estimated speciation (blue, straight line), extinction (red, descending dashed line), and net diversification (purple, ascending dashed line) rates through time for the Terebridae phylogeny. d) RPANDA plot showing the estimated accumulation of species richness through time for the Terebridae phylogeny.

We examined terebrid diversification rates as a function of time and across the six individual Clades A–F delineated in our phylogenic reconstruction (Fig. 2). Using a realistic sampling fraction of 26%, BAMM analysis supported a model that indicated a steady rate of terebrid diversification over time, with a 0.97 posterior probability. Both posterior probabilities and Bayes factors were remarkably lower for alternative models with one or two rate shifts (Supplementary Table S2 available on Dryad). The credible shifts plot depicts a single evolutionary regime for the Terebridae regardless of the value attributed to the prior probability of a rate shift (Fig. 3A and Supplementary Table S2 available on Dryad). The rate-through-time BAMM plot supports a scenario of a slow increase of diversification for the whole Terebridae (Fig. 3B). This scenario is further corroborated by the results of RPANDA analysis, which recovered a rate-constant speciation (lambda = 0.134 lineages/myr) and rate-variable extinction model as best to describe the evolutionary pattern of the Terebridae. More specifically, the extinction rate has decreased over time and the diversification rate has plateaued, according to the best-fit RPANDA model (Fig. 3C). From these analyses, the decrease in terebrid extinction rate can explain an increase in global diversification rate beginning around 25 Ma, as has been observed in other marine taxa (Alfaro et al. 2007; Williams and Duda 2008).

### Evolution Rate Shifts in Depth and Shell Size

Despite the absence of across-clade heterogeneity in diversification rates, the most supported configurations recovered by BAMM analysis for continuous traits displayed evidence of shifts in evolutionary rates of terebrid traits. Specifically, for shell size, we recovered two likely evolutionary rate shifts: one for the single species *Myurellapertusa* belonging to clade E1 and the other for Clades B and C, corresponding to the *Terebra* and *Oxymeris* genera (Supplementary Fig. S6 available on Dryad). Shell size appeared to have undergone a fast divergence at the beginning of the Terebridae evolutionary history, followed by several oscillations between 35 and 15 Ma, with the evolutionary rate still increasing toward the present (Supplementary Fig. S7 available on Dryad). Our sample ranged in length from 10 mm (*Partecosta trilineata*) to 274 mm (*Oxymeris maculata*), with an average length of 61 mm, 104 species were classified as being $>$25 mm and 27 species $\le $25 mm.

Similarly, depth apparently underwent seven shifts in evolutionary rates that are summarized in the four groups outlined (Supplementary Fig. S8 available on Dryad): 1) One shift for a subset of clade C including *Terebra* n. sp. aff. *cumingii* 1 (shallow), *Terebra* n. sp. aff. *cumingii* 2 (deep), *Terebra* n. sp. 27 (shallow) and *Terebra cumingii *(deep). 2) One shift for the subset of Clade E1, which is a shift to deep waters shared by *Myurella brunneobandata, M. pseudofortunei* and M. n. sp. aff. Fortune. 3) Three shifts from shallow to deep for subsets of Clade E2, including, respectively, *Punctoterebrateramachii* and *Punctoterebra baileyi, Punctoterebra polygyrata, P. trismacaria* and *P. textilis*, *P.* sp. aff. *textilis* 1, and *P.* n. sp. aff*. trismacaria* 1. *4.* The last two shifts are in the E5B clade for the species *Myurellopsis joserosadoi* and *Myurellopsis guphilae* were both shifts to deep waters (Supplementary Fig. S8 available on Dryad).

**Figure 4.**
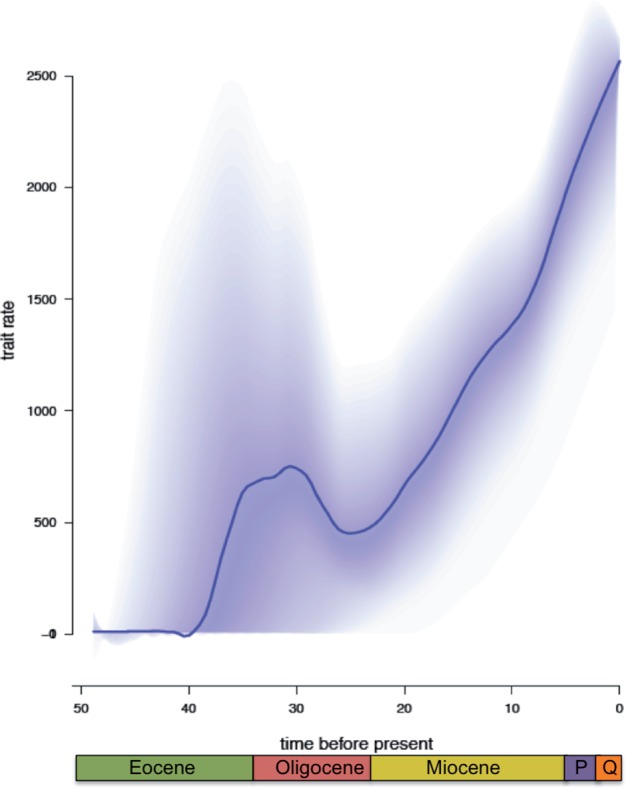
Terebrid depth diversification rate varies over time. Rate vs. time plot from the depth trait BAMM analysis, where “trait rate” is given as depth change per million years, and “time before present” is in millions of years. At the start of terebrid evolution depth trait has a constant diversification rate, then in the Oligocene there is a sharp increase, followed by a decline until approximately 25 Ma, when the depth trait appears to steadily increase continuing into present day.

The rate-through-time plot for depth distribution emphasizes a constant, very low evolutionary rate at the beginning of Terebridae evolutionary history, followed by a steep increase at ca. 40 Ma, a marked decrease after 30 Ma, and a second rapid increase from ca. 25 Ma to the present (Fig. 4). From the specimens used in our data set, certain species, such as *Pellifronia jungi*, which was found 400–780 m over a range of widespread localities, remain in deep waters, whereas other species, such as *Hastula hectica*, remain in shallow waters exhibiting a minimum depth of 0 m and maximum depth of 3 m. One hundred and forty eight species were classified as deep water being found below 100 m and 64 species classified as shallow were found above 100 m. Although most species have a narrow depth range, certain terebrid species have a broad depth range, such as *Myurella nebulosa*, which has a minimum depth of 1 m and maximum depth of 762 m, or *M. joserosadoi* with a minimum depth of 5 m and maximum depth of 287 m.

According to the values retrieved for Pagel’s $\lambda $ (0.91 for both traits), both depth and shell size have a strong phylogenetic signal, indicating that close relatives are more similar to each other for what concerns these traits than to distant relatives (Supplementary Table S5 available on Dryad).

### Redefinition and PD of Terebrid Foregut Anatomy Involving Predation-Related Traits

The presence or absence of a proboscis (PR), VG, odontophore, accessory proboscis structure (APS), and salivary glands (SG), and ranked the type of marginal teeth (RadT; absent, duplex, solid recurved, flat, semi-enrolled, or hypodermic) were evaluated to redefine the feeding types present in 51 of the 199 terebrid species used in this study. We identified 12 unique foregut anatomies (Types I–XII) defined by unique combinations of the six studied characters (Fig. 2 and Table 1). It is important to note our anatomy Types I–XII are distinct from Miller Types I–III (Miller 1971). In our analyses, certain anatomy types are clade specific, such as Type XII, which is only found in the genus *Terebra* (Clade C), whereas other anatomy types can be found in multiple clades, such as Type I, which can be found in *Oxymeris* clade B and in the *Myurella, Punctoterebra, Neoterebra*, and *Maculauger* E subclades. Type XII represents species with both a venom apparatus and APS, suggesting this morphology could be an intermediate between terebrids that have a venom apparatus and those that lack it. The APS is usually found in terebrid and other conoidean species that have lost radula and VG, and even on those occasions it is a seldom occurrence in these families (Fedosov 2007; Fedosov and Kantor 2008). The APS was suggested to have enabled novel feeding strategies which did not involve prey envenomation, or enhanced switch to different prey taxa (Fedosov and Kantor 2008; Holford et al. 2009b). Anatomy Type XI represents the traditional conoidean venom features and is found in terebrids, cone snails, and most other Conoidea lineages. Summarily, the 12 anatomy types identified reflect the substantial degree of plasticity in terebrid foregut.

**Table 1.** Twelve newly defined terebrid anatomy types

			Defining characteristics				
Anatomy type	Species representatives	Clade	Proboscis	VG	SG	APS	Marginal teeth
I	*Oxyermis areolata, Myurella amoena, Punctoterebra solangeae, Neoterebra armillata, Maculauger minipulchra*	B, E1, E2, E4, E5A					Absent
II	*Myurella affinis, Myurellopsis parkinsoni*	E1, E5B				✓	Absent
III	*Neoterebra variegata, Maculauger pseudopertusa*	B, E4			✓		Absent
IV	*Myurellopsis nebulosa, Myurellopsis undulata*	E1, E5B			✓	✓	Absent
V	*Partecosta fuscolutea, Duplicaria bernadii*	F1, F2			✓		Solid recurved
VI	*Punctoterebra succincta*	E2	✓		✓	✓	Absent
VII	*Neoterebra puncturosa*	E4	✓		?	✓	Absent
VIII	*Profunditerebra poppei*	E3	✓	✓	✓		Duplex
IX	*Punctoterebra lineaperlata*	E2	✓	✓	✓		Flat
X	*Hastula stylata*	D	✓	✓	✓		Semienrolled
XI	*Terebra subulata, Hastula hectica, Myurellopsis kilburni*	C, D, E5B	✓	✓	✓		Hypodermic
XII	*Terebra quoygaimardi*	C	✓	✓	✓	✓	Hypodermic

*Notes*: Twelve anatomy types were defined by looking at the presence or absence of a proboscis, VG, SG, or APS, as well as looking at the type of marginal tooth. Species listed do not encompass all species with the anatomy type, but rather a subset, while clades represent all of the clades that contain each of the anatomy types.

Phylogenetic signal and PD analysis with regard to the presence or absence of a VG were carried out on a subset of 51 species. The strong phylogenetic signal ($D={\rm t}1.08$) obtained for the VG indicates that the trait is phylogenetically conserved, indicating that members of a same clade tend to share same trait state. Through a PD analysis, negative SES values and low quantiles were obtained both for the MNTD and for the MPD of the species without a VG, indicating that their phylogenetic distance is smaller than expected (Supplementary Table S6 available on Dryad). These results confirm the conservatism of the trait identified by the phylogenetic signal, and highlight that the loss of a VG happened in phylogenetically clustered terminal taxa, and that when the VG is lost in the ancestor, the reversal is extremely unlikely.

### Distribution and PD of Terebrid Larval Ecology

We examined the protoconch in a total of 638 intact terebrid adult specimens belonging to 116 species. In our data set, multispiral (m) protoconchs had between three and five whorls, and paucispiral (p) protoconchs had a maximum of 2.25 whorls. A number of specimens displayed an intermediate protoconch, with 2.5 whorls and a general appearance compatible either with a lecithotrophic larva with a longer dispersive stage, or a short-lived planktotrophic larva. In those cases, instead of using only whorl numbers, the shell was attributed to one of the two developmental types based on protoconch characteristics, where a small nucleus and an evident boundary between protoconch and teleoconch were considered indicative of a planktotrophic development. Of the 199 species examined in the study, 72% are planktotrophic and 28% are lecithotrophic (Fig. 2 and Supplementary Fig. S9 available on Dryad).

Phylogenetic signal was quite strong for larval development ($D={\rm t} 0.21$), whereas PD analysis recovered negative SES values and low quantiles for MNTD of the lecithotrophic community only. The values obtained for MPD were negative with low quantiles for the planktotrophic community, and positive with high quantiles for the lecithotrophic community (Supplementary Table S6 available on Dryad). The negative MNTD values for the lecithotrophic community indicate that the phylogenetic distance among lecithotrophic species is smaller than expected, and that this clustering can be detected closer to the tips of the phylogeny. Therefore, lecithotrophy appears to be a trait shared by closely related species, indicating that it has evolved before separation of the species-level lineages and supporting the current view that reversal to planktotrophy is an unlikely event. Conversely, the obtained MPD values suggest that PD is high for planktotrophic developers, and indicates a more ancient origin of phylogenetic clustering.

### Evolutionary Modeling of Traits Establishes Larval Development and Shell Size Relationship

We identified an evolutionary link between larval ecology and shell size in the Terebridae using OUwie analyses. Specifically, the rate of shell size evolution is more than five times higher in planktotrophic species (*$\sigma $*$^{2} = 83.15 \pm 0.23$) than in lecithotrophic species (*$\sigma $*$^{2} = 15.1 \pm 0.51$), whereas the strength of pull toward a shell size optimum is about three times higher for lecithotrophic species ($\alpha = 0.30 \pm 0.043$) than for planktotrophic ones ($\alpha = 0.67 \pm 0.01$). This finding is based on the best-fitting model for the Terebridae adult shell size across the species included in our data set, which is the OUMVA, according to the Akaike weights, with a delta AICc $>$5 with respect to the second best-fitting model OUMA (Supplementary Table S7 available on Dryad). This model allows the larval ecology to influence the optimal shell size, the rate of shell size evolution and the strength of pull toward the optima across our Terebridae data set. The optimal shell size value itself ($\theta$) has a value of 70 ($\pm $18) mm for planktotrophic and 21 ($\pm $7) mm for lecitotrophic species. Our results suggest that species with long-living pelagic larvae not only tend to be generally larger, but also have a wider shell size range than lecitotrophic species. The best-fitting model for depth distribution was a simple Brownian model (BM), which did not support any correlation between depth and larval development. Likewise, when coded as a discrete trait, there was no support for a correlation between shell size and depth distribution.

### No Clear Drivers of Terebrid Diversification

Potential key innovations such as venom apparatus, larval development, shell size, and depth distribution were examined in BiSSE using several models of trait evolution to determine potential drivers of terebrid diversification (Supplementary Table S4 available on Dryad). Contrary to our expectations, for presence or absence of VG, the best-fit model had irreversible transition rates and equal speciation and extinction rates, suggesting the presence or absence of the VG does not impact the rate of diversification in the terebrids. For larval development, shell size, and depth, we recovered the same speciation, extinction, and transition rates for the two trait states considered, thus detecting no significant departure from the null model. These results do not identify any of the tested traits as drivers of diversification, suggesting that either additional traits and/or sampling of species is required, or terebrid diversification is not driven by a key innovation but rather by ecological opportunity due to environmental conditions.

## Discussion

A robust dated phylogenetic reconstruction of predatory terebrid marine snails was used as a framework for investigating the influence of several potential factors, such as anatomical features linked to the venom apparatus, larval development, shell size, and depth distribution, on terebrid species diversification.

The molecular phylogeny presented here is based on a significant increase in the taxonomic coverage compared with previously published phylogenies for the group, tripling the number of specimens used and almost doubling the number of species. Specifically, the number of specimens sequenced increased from 406 used in the previous terebrid phylogeny (Castelin et al. 2012) to 1275 in the current study. This sampling increase corresponds to about 40% of the $>$400 described species, which is 26% of the estimated species diversity, and further confirms the monophyly of the family Terebridae and the existence of six major clades (Clades A–F; Holford et al. 2009b; Castelin et al. 2012).

In our molecular phylogenetic analysis, Clade F (including genera *Duplicaria* and *Partecosta*) has a new position and is recovered as a sister group to all other terebrids. In prior publications, *Pellifronia* clade A was found to be the sister group to all other terebrids (Castelin et al. 2012; Modica et al. 2014; Fig. 2). This shift could be explained be the addition of *Bathyterebra coriolisi* in Clade A, which is a very different species compared with *P. jungi*, the only species in the Castelin et al. (2012) analyses. We also identified a number of pseudo-cryptic species within species complexes, which suggest that a considerable fraction of the diversity in the Terebridae still needs formal description (Fedosov et al. 2019). Although the overall topology of terebrid phylogeny did not change significantly, adding more samples helped to reveal species complexes and to increase the overall node support, illustrating the importance of dense species sampling to more accurately reconstruct phylogenies.

### Diversification Is Constant Across Clades, and Slowly Increasing Across Time in Terebridae

The results obtained by BAMM analysis of terebrid diversification rates across clades outlined the absence of any clade-specific shift in diversification rates. In contrast, the diversification rate-through-time plot obtained in BAMM suggests that the diversification rate is slowly increasing in the Terebridae, when using a sampling fraction of 26% of total extant terebrid diversity (Fig. 3B). The shape of the rate-through-time plot suggests that diversification rates were increasing faster at the roots of the Terebridae phylogenetic tree, and tend to slow down closer to the present while still increasing. These results were corroborated by RPANDA analysis that also highlighted that the increase in diversification rates can be attributed to a decrease in extinction rate starting about 25 Ma (Fig. 3C).

The lack of clade-specific diversification rate shifts was unexpected given the uneven species richness and anatomical disparity observed in different clades. The relationship between species richness and diversification rates has been intensely debated, and it is presently generally accepted (McPeek and Brown 2007; Rabosky 2009; Wiens 2011; Rabosky et al. 2012). The strength of this relationship has been demonstrated to be rather variable, and may be lowered by several factors including negative age–diversification relationships in which younger clades tend to evolve faster than older clades (Kozak and Wiens 2016; Scholl and Wiens 2016). This pattern may be explained by density-dependence slowing diversification rates over time, or by the younger clades having novel traits that can help explain their rapid diversification (Rabosky 2009; Wiens 2011; Scholl and Wiens 2016). In addition, homogeneity of across-clade diversification has been explained in some cases by the acquisition of a group-wide key innovation, which lead to a constant diversification rates, as is the case with pharyngeal jaws in labrid fishes, climbing behavior in woodcreepers, and locomotion strategies in triggerfish (Alfaro et al. 2009; Dornburg et al. 2011; Claramunt et al. 2012). In some circumstances, diversification rates have been even shown to decrease after the acquisition of such key innovations, as evidenced by the development of foregut fermentation in colobine monkeys (Tran 2014).

### Foregut Anatomy and Ecological Traits Are Not Drivers for Terebrid Diversification

Our results suggest that trait evolution in morphological and ecological traits is not linked to terebrid diversification. Using a BiSSE analysis, none of the traits examined, venom apparatus, larval development, bathymetric distribution, and shell size, was identified as key innovations able to affect Terebridae diversification rates. The finding that foregut anatomy did not have any effect on diversification rates was surprising given the uneven species richness observed across lineages with different foregut anatomies. This is particularly relevant for the VG in the foregut as the production of venom has been proposed as a key innovation driving diversification in Conoidea (Castelin et al. 2012) and in other venomous taxa such as snakes (Vidal and Hedges 2005; Fry et al. 2006), as it can allow the exploitation of new prey types and thus the colonization of novel niches. Our results, however, agree with a recent work, which reported the presence of a VG had no effect on diversification rates across the conoidean tree (Abdelkrim et al. 2018).

It should be noted that the venom apparatus, which consists of a VG, hyperdermic radular teeth, and proboscis, is a shared evolutionary novelty of most lineages of the Conoidea and is lacking in some terebrids. In other words, in some clades of the Terebridae the loss of the venom apparatus and not its acquisition is observed, for example, in the entire *Oxymeris* clade (Clade B). BiSSE best-fit model supported the hypothesis, already proposed on anatomical basis, that the loss of the VG is irreversible and this was also corroborated by the PD results. It is unclear how these species can effectively predate, but the evidence of increased abundance of terebrid species with no VG, compared with those retaining a venom apparatus within a given area or locality seems to suggest that this loss does not imply any selective disadvantage (Kantor et al. 2012; Fedosov et al. 2014). This finding is confirmed by a recent stable isotope study investigating feeding habits of the Terebridae in which the ranges of trophic niches were indistinguishable between lineages with a venom apparatus and those without (Fedosov et al. 2014). In addition, venom components were reported in foregut glands such as the SG, which are not considered as part of the venom apparatus, suggesting that, as in other venomous gastropods, even those Terebridae lineages that lack a venom apparatus may still produce bioactive compounds that can be released into the water to subdue prey (Modica et al. 2015; Gerdol et al. 2018). These observations, along with the finding that neither the loss nor the acquisition of a venom apparatus influence diversification rates in Terebridae, imply that venom apparatus is not, by itself, a good indicator of selective advantages linked to trophic ecology. Other feeding-related traits such as SG or general biochemical venom diversification may reveal better proxies of trophic adaptation.

### Colonization of Deep Waters May Have Affected Overall Terebridae Diversification

The observed lack of support for clade-specific terebrid diversification rate shifts, suggests the overall increase in diversification rate affecting the family may be due to a group-wide factor, rather than to traits displaying a high level of lineage-specific disparity. A potential hypothesis to explain the generalized increase in diversification rates across the entire terebrid family is an ecological release initiated by the colonization of deep waters. A constant increase in diversification rates was identified in bird genus *Grebes* and was hypothesized to be caused by fragmentation of habitat, a factor that affected the entire family (Ogawa et al. 2015). Similarly, a study focused on freshwater snails showed an increase in speciation rates after experiencing ecological opportunity through dispersal to new locations (Delicado et al. 2018). For Terebridae, we retrieved a BAMM rate-through-time plot of depth distribution characterized by a constant and low diversification rate at the beginning, followed by a steep increase at about 40 Ma, a decrease at 30 Ma, and a second rapid increase in diversification rates from about 25 Ma to the present (Fig. 4). Therefore, we propose a scenario wherein terebrids, after having originated in shallow waters, increased their depth range by moving with a set of adaptions that progressively allowed them to reposition at deeper zones when sea levels began to fall. This led them to colonize new niches, where selective pressure due to competition and predation were weaker, which enabled a slow, but steady increase of diversification due to the reduction of extinction rate. The conquest of deep-sea habitats may have been triggered by an increase of sea level, which reached a maximum in the early Eocene ($\sim $50 Ma; Miller et al. 2005; Kominz et al. 2008). At the same time, the increase in sea levels may have contributed to lower the extinction rates through the increase of the continental shelf surface and, therefore, an increase in habitats (Orzechowski et al. 2015). Both the time estimates for main increase of depth diversification rate retrieved from BAMM and the paleontological dating of Eocene sea-level rise match with the time corresponding to lowest estimate of the extinction rate found in RPANDA analysis (Fig. 3C). As sea levels began to fall, extinction rates in the Terebridae began to level off ($\sim $30 Ma). Therefore, similar to the “colonization of deep waters” hypothesis, the availability of more habitats created by the increased sea level would have contributed to an ecological release through a decrease in competition for resources on the shelf. The mosaic of habitat types in the Indo-West Pacific, a diversity hotspot for Terebridae as well as for other marine invertebrates, might have contributed to ecological release, as already suggested for other gastropod taxa (Williams and Duda 2008).

The lack of statistical support for this hypothesis from BiSSE modeling may be due to insufficient taxonomic coverage. In fact, simulation studies suggested that BiSSE modeling performs best with $>$300 terminal taxa (Davis et al. 2013; Gamisch 2016). Despite the three-fold increase with respect to previous phylogenies, our data set still represents merely 26% of estimated Terebridae diversity. In addition, our sampling effort has been mostly concentrated on less known deep-water habitats, leading to a potential overrepresentation of deep-water species in our data set. We recognize that our deep water sampling bias may not reflect the actual distribution of Terebridae diversity, and may have affected the results of trait evolution modeling.

### Larval Development Affects Terebrid Adult Shell Size

Notably, for the first time we demonstrate that lecithotrophy has evolved at least 18 times in the Terebridae and there is a link between adult shell size and larval development. We corroborate in the terebrids, as in previous studies on other gastropod taxa, that larval development evolution trends are generally unidirectional, moving from planktotrophy to lecithotrophy (Gould and Eldredge 1986; Rouse 2000; Collin et al. 2007). In the Terebridae, shell size appears to follow a complex history of diversification. Across our entire data set, the best-fitting model estimates with strong support, according to Akaike weights, a different optimal size for the two divergent larval ecologies but with a higher strength of pull toward a size optimum in the lecithotrophic species. In detail, this model consistently estimates that adult size in lecithotrophic species is significantly smaller, and more strictly size-constrained, than in planktotrophic species, despite a larger egg size, which in turns determines the appearance of the protoconch. This implies that size in later stages of life is mostly linked to the length of the larval stage (Levin et al. 1987; Miller and Hadfield 1990; Havenhand 1993). The increased shell size in planktotrophic terebrids could be derived from longer generation times, which has been discussed in the settlement-timing hypothesis (Todd and Doyle 1981). A pelagic larval development is displayed by the vast majority (ca 70%) of marine invertebrate species, and is considered the ancestral larval ecology in gastropods (Thorson 1950; Nielsen 2009), including most lineages of Caenogastropoda (Haszprunar 1988). The dichotomy between the two contrasting larval ecologies has been well studied in marine invertebrates: planktotrophic species have smaller egg sizes and high female fecundity and lecithotrophic species possess lower female fecundity and larger egg sizes, and they can, therefore, be placed at the two edges of an r-K continuum (Thorson 1950; Vance 1973; Strathmann 1977; Todd and Doyle 1981). As lecithotrophic terebrid species rely on yolk reserves that are fixed at the moment of egg production, their shell size at the time of hatching is fixed, whereas in planktotrophic species it may vary according to the length of larval stage and food intake. Thus, it may be argued that the shift to lecithotrophy, with its stronger constraint on adult shell size, may reduce the plasticity of species and their ability to adapt to new niches, partly explaining why the acquisition of lecithotrophy, despite leading to a reduction of interpopulational gene flow, does not increase speciation rate. The link between adult shell size and larval development we have identified in terebrids may upon examination also be present in other families of marine gastropods.

## Conclusions

Identifying the factors that influence predator–prey interactions and macroevolutionary patterns that lead to species diversification remains a challenge in neglected marine invertebrates. In this study, we examined the Terebridae, an understudied group of predatory sea snails that possess a notable range of foregut anatomical features and a complexity of venom arsenals comparable with other groups of the Conoidea (Imperial et al. 2007; Kendel et al. 2013; Anand et al. 2014; Gorson et al. 2015; Eriksson et al. 2018). Despite a long-standing hypothesis that venom can be a driver for diversification, we did not find a correlation between possession of a venom apparatus and terebrid diversification. This is a remarkable difference from what is reported in advanced snakes (Vidal 2002; Fry et al. 2008; Pyron and Burbrink 2012) and venomous lizard lineages (Fry et al. 2006). However, our results are in agreement with recent findings that the presence of a VG does not significantly affect diversification rates across the conoidean tree of life (Abdelkrim et al. 2018). Although larval development did not appear to play a role in the diversification of Terebridae, evolutionary modeling identified a strong link between larval ecology and variability of adult shell size, highlighting larval ecology as an indirect factor shaping the Terebridae evolutionary trajectory. Our results indicate that environmental factors linked to past sea level increase and depth range may have played a key role in terebrid diversification, acting as major evolutionary drivers on the whole family. The terebrids are a microcosm for understanding diversification within marine mollusks, and our findings are an important milestone in disentangling true drivers of evolutionary success that lead to the astounding speciation of this group and in the family Conoidea.

## Supplementary Material

Data available from the Dryad Digital Repository: http://dx.doi.org/10.5061/dryad.j008j5h.
